# Neighbourhood characteristics related to mental health service use among adults with diabetes: a population-based cohort study in New Brunswick, Canada

**DOI:** 10.1186/s13104-022-05966-9

**Published:** 2022-02-23

**Authors:** Neeru Gupta, Dan Lawson Crouse, Ismael Foroughi

**Affiliations:** 1 Department of Sociology, University of New Brunswick, PO Box 4400, Fredericton, NB E3B 5A3 Canada; 2 Health Effects Institute, Boston, USA

**Keywords:** Diabetes mellitus, Mental disorders, Social determinants of health, Population health, Environment design, Residence characteristics, Public health surveillance, Syndemic

## Abstract

**Objective:**

It has been postulated that social and economic inequalities may shape the distributions of comorbid diabetes and mental illness. This observational cohort study using linked population-based administrative and geospatial datasets aimed to describe associations between neighbourhood socioenvironments and disorder-specific mental health service use among adults with diabetes in the province of New Brunswick, Canada.

**Results:**

A baseline cohort of 66,275 persons aged 19 and over living with diabetes was identified. One-quarter (26.3%) had used healthcare services for mood and anxiety disorders at least once during the six-year follow-up period 2012/2013–2017/2018. Based on Cox proportional hazards models, the risk of mental health service contacts was significantly higher among those residing in the most materially deprived neighbourhoods [HR: 1.07 (95% CI: 1.01–1.14)] compared to those in the least so, and those in areas characterized with the highest residential instability [HR: 1.13 (95% CI: 1.05–1.22)] compared to those in areas with the lowest instability. Among adults with incident diabetes (N = 4410), age and sex but not neighbourhood factors were related to differential help-seeking behaviours for mental health problems. These findings underscored the gap between theoretical postulations and population-based observations in delineating the syndemics of neighbourhood socioenvironments and mental health outcomes in populations with high diabetes prevalence.

## Introduction

Diabetes mellitus is often accompanied by other health problems, which may result in poorer health-related quality of life and greater use of healthcare services versus those living with diabetes alone [1–4]. Rising incidence and prevalence of diabetes (type 1 and type 2) worldwide is fuelling the need for evidence-informed interventions to prevent or delay common comorbidities at the population level [5–8]. A growing body of literature is enumerating the mental health implications of the experience of living with diabetes, including higher co-occurrence of depression and other mood and anxiety disorders [9, 10]; however, much of the research has focused on the clinical and epidemiological aspects, with limited attention to the social forces that may contribute to the onset of adverse mental health outcomes among persons with chronic disease [11].

It has been postulated that social and economic inequalities can shape the distributions and exacerbate the syndemic clusterings of comorbid diabetes and mental illness within and across populations [12–15]. Observational studies from different contexts have found neighbourhood socioeconomic disadvantage and less favourable walking environments to be independently associated with diabetes incidence and prevalence [16–18]; the associations of neighbourhood characteristics with mental health disorders and related service use are seemingly less robust [19, 20]. The interaction effects of neighbourhood environments and different health and health system metrics remain complex and the causal pathways are less well understood [21], particularly in populations characterized by smaller urban and rural communities [22]. A recent systematic review highlighted the knowledge gap in having identified a single study quantifying the associations between neighbourhood disadvantage, severe depression, and type 2 diabetes risk [23], with little evidence of synergistic interaction emerging from the reviewed Swedish sample [24].

In research elsewhere, we found only selected socioenvironmental characteristics of local communities were associated with increased mental health service use among older adults surviving myocardial infarction in New Brunswick, one of Canada’s most rapidly aging and most rural provinces [25]. A need for further research to better understand the role of community situations to improve mental health outcomes in populations with high chronic disease burden was identified. For this study, we extend the analysis to uniquely test for associations between neighbourhood factors and mental health service use among all adult New Brunswickers with pre-existing or newly diagnosed diabetes (types 1 or 2). Our aim is to provide insights into the modifying role of neighbourhood socioenvironments on incident disorder-specific mental health service contacts among adults with diabetes in this context of universal medical coverage. Specifically, a population-based observational cohort analysis was conducted, using linked administrative and geospatial datasets to assess the risk of service use for mood and anxiety disorders among patients with diabetes over the period 2012/2013–2017/2018.

## Main text

### Materials and methods

#### Study setting

Located in eastern Canada, the semi-rural province of New Brunswick is characterized with high rates of diabetes and of mental health service use. Diabetes prevalence stood at 10.7% and incidence at 0.8% in 2016, both rates significantly higher than the national averages (8.8% and 0.6% respectively) [26]. Meanwhile, 10.9% had used healthcare services for a mood or anxiety disorder in the same year, slightly above the national average (10.3%) [26]. The provincial diabetes prevention and control strategy identified the need to address comorbid mental health challenges as well as interactions among social and economic factors [27], although associated baseline measures and benchmarks for use in practice and research to inform sustainable investments were lacking, underscoring the need for improved evidence.

#### Study design and target population

Following research approaches detailed elsewhere [25], we linked longitudinally multiple person-level provincial administrative health datasets with area-based socioenvironmental datasets for the population of New Brunswick, Canada. Four pseudonymized administrative datasets were used: resident registrations for public healthcare insurance, vital statistics, annual case ascertainments for diabetes (types 1 and 2 combined), and annual ascertainments for the use of healthcare services for mood and anxiety disorders. Record linkages were performed deterministically using patients’ (scrambled) insurance numbers. Given the context of universal medical insurance, the data captured virtually all physician and hospital services among all residents, and this according to age, sex, and place of residence. Based on annual residential postal code information, each individual was assigned a series of neighbourhood-level indicators of socioenvironmental characteristics from datasets made available through the Canadian Urban Environmental Health Research Consortium [28]. The data were accessed in the secure computing environment of the New Brunswick Institute for Research, Data and Training (NB-IRDT) [29].

The study cohort comprised all adults 19 years and over and residing in New Brunswick ever diagnosed or newly diagnosed with diabetes in the baseline fiscal year of 2012/2013. These individuals were then followed over six years of observation, that is, to the end of the 2017/2018 fiscal year. Patients who died or moved away from the province during this period were censored. The case ascertainments were tallied using an algorithm for identifying population-based diabetes prevalence and incidence from administrative health data in accordance with validated data standards of the Canadian Chronic Disease Surveillance System (CCDSS) [8, 30]. Since type 1 diabetes is most often diagnosed in childhood and adolescence [7], incident cases among the adult population are assumed to chiefly reflect type 2 diabetes.

The outcome of interest was patients’ use of medical or hospital services at least once in a given year for a mood disorder (e.g., depression, neurosis, affective psychosis), an anxiety disorder (e.g., social phobia, panic disorder, dream anxiety disorder), or both. Ascertainments of service use for mood and anxiety disorders based on administrative information drew on validated CCDSS case definitions [31, 32].

#### Neighbourhood characteristics

The mental health implications for five different indicators of neighbourhood socioenvironments among persons with diabetes were considered. These included four area-based composite indicators of social and economic inequality collated in the Canadian Marginalization Index: material deprivation (e.g., proportion of low-income families, unemployment, homes needing major repair); residential instability (e.g., level of crowding, residential ownership, residential mobility); ethnic concentration (proportions of recent immigrants and visible minorities); and population dependency (e.g., labour force participation, proportion of seniors) [28, 33]. The fifth socioenvironmental indicator was derived from the Canadian Active Living Environments dataset, a summary index of features of communities that support active living, such as densities of homes, parks, footpaths, and transit stops [34]. The geocoded measures were ranked into nationally standardized groupings for New Brunswick’s 1454 census dissemination areas.

#### Statistical analysis

Cox proportional hazards regression analysis was used to assess the associations between health service contacts for mood and anxiety disorders and neighbourhood characteristics among the adult population with prevalent or incident diabetes. To control for recent history of common mental disorders, patients having used mental health services in the 3 years preceding baseline (that is, based on retrospective data from 2009/2010 to 2011/2012) were excluded from the analysis. Individuals’ age was included as a time-varying confounding factor over the period of observation and sex as a time-invariant confounder in the models. Adjusted hazard ratios (HRs) and bootstrapped 95% confidence intervals (CIs) were generated for each predictor using Stata v15 statistical software. Population counts were rounded to a base of five to reinforce the confidential nature of the administrative health data. We followed the RECORD (REporting of studies Conducted using Observational Routinely collected health Data) guidelines in our reporting [35].

## Results

### Study population

Among the New Brunswick population aged 19 and over at baseline (N = 621,385 in 2012/2013), 71,560 had been diagnosed with diabetes mellitus. After excluding individuals with a recent history of prevalent mental disorders (N = 2305) and those without complete residential history information in the province over the 6-year follow-up period (N = 2980), the cohort for analysis included 66,275 adults with diabetes residing in one of 1374 neighbourhoods. The subcohort of those newly diagnosed with diabetes in the year counted 4410 individuals.

### Descriptives

**Fig. 1:**
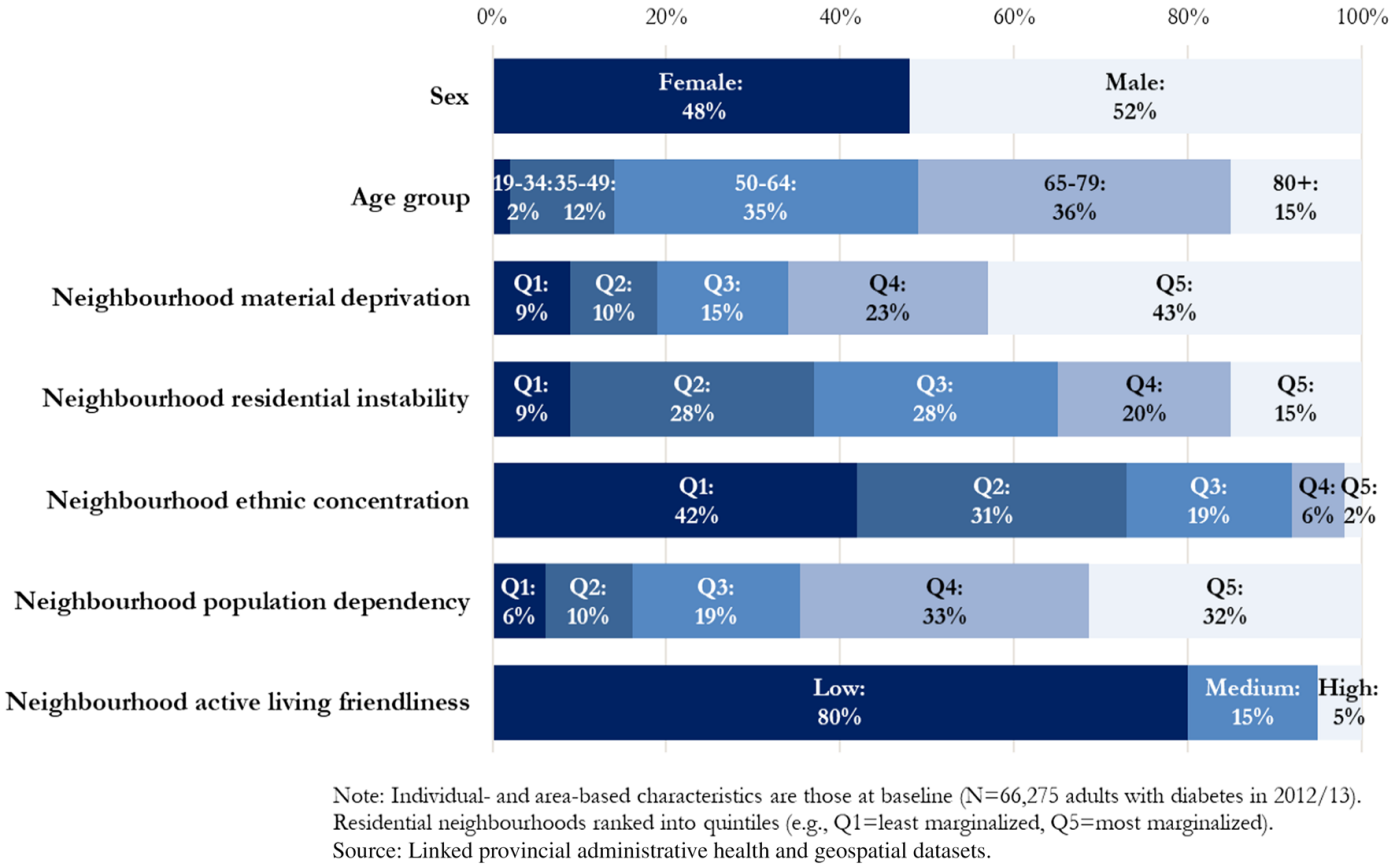
Percentage distribution of the adult population with diabetes by individual and neighbourhood-level characteristics, New Brunswick, Canada

Of the cohort of adults with diabetes, at least two-thirds were residing in neighbourhoods characterized by higher material deprivation (quintiles 4–5), higher population dependency, and low active living friendliness (Fig. 1). In the New Brunswick context, relatively few were residing in areas characterized by high ethnic concentration (8% in quintiles 4–5), as expected based on research findings elsewhere on the province’s adult population with chronic disease [25].

Over the 6-year period of observation, 26.3% of the study population had used healthcare services at least once for a mood or anxiety disorder; the proportion was 19.3% among adults with newly diagnosed with diabetes (Table 1). Usage rates were higher among women than men and among younger adults than seniors, echoing results from our earlier investigation of mental health service contacts among adults with cardiac disorders [25]. While rates were somewhat higher among those residing in neighbourhoods characterized with greater material deprivation, the directions and magnitudes of the relationships between mental health service use and the various neighbourhood factors were less clear compared with those observed for individual demographics.

**Table 1:** Percent of the adult population with diabetes having used healthcare services for a mood or anxiety disorder, by individual and neighbourhood-level characteristics

Characteristic	(1) All adults with diabetes (N = 66,275 prevalent cases) (%)	(2) Newly diagnosed diabetes (N = 4410 incident cases) (%)
Sex		
Female	32.5	23.9
Male	20.8	15.9
Age group		
19–34 years	37.9	28.6
35–49 years	35.6	24.0
50–64 years	29.4	19.0
65–79 years	23.1	17.3
80 years and over	17.6	15.4
Neighbourhood material deprivation		
Quintile 1—least deprivation	24.5	18.1
Quintile 2	25.5	17.4
Quintile 3	26.3	19.9
Quintile 4	26.4	18.1
Quintile 5—most deprivation	26.9	20.4
Neighbourhood residential instability		
Quintile 1—least instability	26.0	21.4
Quintile 2	25.7	18.4
Quintile 3	25.4	18.9
Quintile 4	27.0	18.2
Quintile 5—most instability	28.7	21.9
Neighbourhood ethnic concentration		
Quintile 1—least ethnic	25.3	18.8
Quintile 2	26.8	21.4
Quintile 3	27.4	16.2
Quintile 4	26.8	20.4
Quintile 5—most ethnic	30.0	22.2
Neighbourhood population dependency		
Quintile 1—least dependency	26.9	17.9
Quintile 2	27.9	20.4
Quintile 3	25.9	18.7
Quintile 4	26.1	17.6
Quintile 5—most dependency	26.2	21.4
Neighbourhood active living friendliness		
Low	26.0	18.9
Medium	27.0	19.2
High	29.6	23.1
Total	26.3	19.3

Individual- and area-based characteristics are those at baseline. Data refer to any use of medical or hospital services for mood and anxiety disorders over a 6-year follow-up period

Source: Linked provincial administrative health and geospatial datasets

### Risk of mental health service contacts

Results from the proportional hazards models showed significantly increased risk of using health services for mood and anxiety disorders among adults ever diagnosed with diabetes residing in neighbourhoods characterized by the greatest material deprivation compared with their more affluent counterparts (HR: 1.07 [95% CI: 1.01–1.14], p < 0.05), and among those residing in the most residentially unstable areas compared with the least unstable areas (HR: 1.13 [95% CI: 1.05–1.22]), after adjusting for age and sex (Table 2, model 1). Other observed associations between characteristics of local environments and mental health service contacts among prevalent diabetes cases were mostly not statistically significant. Among the adult population with incident diabetes, none of the neighbourhood-level indicators—only individuals’ demographics—exercised a discernible influence on the outcome variable (Table 2, model 2).

**Table 2:** Adjusted hazard ratios (HRs) and 95% confidence intervals (CIs) for associations between individual and neighbourhood-level characteristics and risk of healthcare use for a mood or anxiety disorder among adults with diabetes

Characteristic	(1) All adults with diabetes (N = 66,275)			(2) Newly diagnosed diabetes (N = 4410)		
	HR	95% CI	*p*-value	HR	95% CI	*p*-value
Sex (ref: Male)						
Female	1.71*	1.66–1.77	< 0.01	1.60*	1.39–1.83	< 0.01
Age group (ref: 35–49 years)						
19–34 years	1.00	0.91–1.10	0.99	1.17	0.80–1.71	0.41
50–64 years	0.80*	0.77–0.84	< 0.01	0.74*	0.61–0.90	< 0.01
65–79 years	0.60*	0.57–0.63	< 0.01	0.63*	0.52–0.77	< 0.01
80 years and over	0.42*	0.39–0.44	< 0.01	0.54*	0.41–0.71	< 0.01
Neighbourhood material deprivation (ref: Quintile 1)						
Quintile 2	1.03	0.96–1.11	0.37	0.94	0.68–1.30	0.71
Quintile 3	1.06	0.99–1.13	0.10	1.12	0.84–1.51	0.44
Quintile 4	1.06	0.99–1.13	0.09	1.01	0.75–1.36	0.93
Quintile 5	1.07*	1.01–1.14	0.03	1.13	0.85–1.50	0.39
Neighbourhood residential instability (ref: Quintile 1)						
Quintile 2	1.00	0.94–1.06	0.87	0.86	0.67–1.10	0.23
Quintile 3	0.99	0.93–1.05	0.69	0.89	0.69–1.15	0.38
Quintile 4	1.06	0.99–1.13	0.09	0.83	0.63–1.11	0.21
Quintile 5	1.13*	1.05–1.22	< 0.01	0.93	0.66–1.31	0.68
Neighbourhood ethnic concentration (ref: Quintile 1)						
Quintile 2	1.05*	1.01–1.09	0.01	1.17	1.00–1.38	0.06
Quintile 3	1.05*	1.00–1.10	0.05	0.81	0.65–1.01	0.07
Quintile 4	0.98	0.91–1.05	0.57	0.96	0.68–1.35	0.81
Quintile 5	1.00	0.90–1.12	0.93	0.85	0.50–1.44	0.54
Neighbourhood population dependency (ref: Quintile 1)						
Quintile 2	1.07	0.99–1.16	0.07	1.19	0.84–1.68	0.34
Quintile 3	0.99	0.99–1.07	0.85	1.01	0.72–1.42	0.95
Quintile 4	1.04	1.00–1.11	0.34	0.95	0.67–1.34	0.75
Quintile 5	1.05	1.00–1.13	0.24	1.18	0.82–1.68	0.37
Neighbourhood active living friendliness (ref: Low)						
Medium	1.01	0.96–1.06	0.67	1.04	0.83–1.30	0.76
High	1.04	0.97–1.13	0.27	1.30	0.91–1.84	0.15

*p < 0.05; ref = reference category. Data refer to any use of medical or hospital services for mood and anxiety disorders over a six-year follow-up period

Source: Linked provincial administrative health and geospatial datasets

## Discussion

This novel study assessed the role of neighbourhood environments on mental health comorbidities among adults with diabetes in a publicly funded healthcare system, an underexplored area in terms of bridging the gap between theoretical postulation and population-based observation. Drawing on linked province-wide administrative and geospatial datasets tracking individuals’ healthcare service contacts over a 6-year follow-up period, and controlling for prior record of mental disorders, only partial associations were found between selected socioenvironmental characteristics of local communities and the risk of healthcare use for mood and anxiety disorders among adults with prevalent diabetes in the Province of New Brunswick, Canada. Notably, the risk of mental health service contacts was significantly higher among those residing in the most materially deprived neighbourhoods (HR: 1.07 [95% CI: 1.01–1.14]) compared to those in the least deprived areas, and among those residing in neighbourhoods characterized with the highest degree of residential instability (HR: 1.13 [95% CI: 1.05–1.22]) compared to those in areas with the lowest instability. No significant associations were found when distinguishing the analysis among adults with incident diabetes, that is, beyond the biodemographic variables of age and sex.

Although there is much evidence of benefits to physical health of favourable neighbourhood socioenvironments, the present findings are generally consistent with observational studies elsewhere reporting limited evidence of clearly convincing associations between mental health outcomes with neighbourhood characteristics in aging populations. Previous small-area studies based on large datasets have found no significant associations between depressive symptoms and other mood or anxiety disorders with active living environments in Canada [20], between mental disorders with neighbourhood income rank in selected Chinese cities [19], or between depression and type 2 diabetes risk with differing levels of neighbourhood socioeconomic deprivation in Sweden [24]. An investigation from Brazil indicated some geographic clustering of depression and diabetes, but at the (large-scale) state level, which may have reflected heterogeneity in access to diagnostic services [15]. Our study adds to the nascent research into neighbourhood effects on comorbid diabetes and mental illness [23], and reinforces the need for more empirical examinations on the syndemics of neighbourhood socioenvironments and mental health implications in populations with high diabetes burden.

### Limitations

Some limitations to this investigation are noted, including ones inherited from our data sources and linkages as originally applied to the older adult population in New Brunswick presenting with a different cardiometabolic condition [25]. Firstly, it is likely the prevalence of mental health service use was underestimated, since the administrative datasets excluded information on service use from exclusively community-based settings or private practices. In addition, we lacked linkable individual-level data on lifestyle behaviours and socioeconomic characteristics potentially influencing health outcomes. Specific to this enquiry, the CCDSS methodology used here for estimating the population with diabetes from administrative medical and hospital records does not distinguish between types 1 and 2 of the disease, which differ in etiology and healthcare responses [36]. More broadly, the generalizability of results from our study context—one characterized by uniquely smaller urban and rural settlements—is uncertain.

## Data Availability

The datasets used in this study are not readily available because restrictions apply to the accessibility of these confidential data, which were used under license for the current study. Requests to access the datasets for research purposes should be directed to the NB-IRDT (www.unb.ca/nbirdt). Requests for accessing the geocoded datasets of socioenvironmental indices may be directed to the Canadian Urban Environmental Health Research Consortium (www.canue.ca).

## Abbreviations

CCDSS: Canadian Chronic Disease Surveillance System
CI: Confidence interval
HR: Hazard ratio
NB-IRDT: New Brunswick Institute for Research, Data and Training
